# Effects of Parental Omega-3 Fatty Acid Intake on Offspring Microbiome and Immunity

**DOI:** 10.1371/journal.pone.0087181

**Published:** 2014-01-29

**Authors:** Ian A. Myles, Nathan B. Pincus, Natalia M. Fontecilla, Sandip K. Datta

**Affiliations:** Bacterial Pathogenesis Unit, Laboratory of Clinical Infectious Diseases, National Institute of Allergy and Infectious Diseases, National Institutes of Health, Bethesda, Maryland, United States of America; Max Delbrueck Center for Molecular Medicine, Germany

## Abstract

The “Western diet” is characterized by increased intake of saturated and omega-6 (n−6) fatty acids with a relative reduction in omega-3 (n−3) consumption. These fatty acids can directly and indirectly modulate the gut microbiome, resulting in altered host immunity. Omega-3 fatty acids can also directly modulate immunity through alterations in the phospholipid membranes of immune cells, inhibition of n−6 induced inflammation, down-regulation of inflammatory transcription factors, and by serving as pre-cursors to anti-inflammatory lipid mediators such as resolvins and protectins. We have previously shown that consumption by breeder mice of diets high in saturated and n−6 fatty acids have inflammatory and immune-modulating effects on offspring that are at least partially driven by vertical transmission of altered gut microbiota. To determine if parental diets high in n−3 fatty acids could also affect offspring microbiome and immunity, we fed breeding mice an n−3-rich diet with 40% calories from fat and measured immune outcomes in their offspring. We found offspring from mice fed diets high in n−3 had altered gut microbiomes and modestly enhanced anti-inflammatory IL-10 from both colonic and splenic tissue. Omega-3 pups were protected during peanut oral allergy challenge with small but measurable alterations in peanut-related serologies. However, n−3 pups displayed a tendency toward worsened responses during *E. coli* sepsis and had significantly worse outcomes during *Staphylococcus aureus* skin infection. Our results indicate excess parental n−3 fatty acid intake alters microbiome and immune response in offspring.

## Introduction

The modern ‘Western diet’, characterized by increased intake of saturated dietary fat and refined sugar, is correlated with inflammatory and immune-mediated diseases [1]. One of the mechanisms by which the Western diet is thought to contribute to inflammatory disorders is through an excess intake of omega-6 polyunsaturated fatty acids (n−6 PUFA) compared to omega-3 (n−3) PUFA [2], [3]. PUFA influence the inflammatory response, with n−6 PUFA generally associated with pro-inflammatory effects [3], [4] and n−3 PUFA generally associated with anti-inflammatory effects [3]–[5]. This raises the possibility of dietary supplementation with n−3 PUFA, such as in fish oil, as an effective treatment for inflammatory diseases [3], [4], [6], [7]. Although more research is needed to draw clear conclusions, there is evidence that dietary n−3 PUFA may have beneficial effects on a variety of conditions with inflammatory components, such as atherosclerosis and cardiovascular disease [4], inflammatory bowel diseases [3], and allergic diseases [2]. Furthermore, there is evidence that n−3 PUFA are important during development, and that maternal intake during pregnancy protects against the development of allergic and inflammatory disease in infants and children [2] and improves pregnancy outcomes [8].

Omega-3 PUFA may modulate the immune response through several potential mechanisms. Increased n−3 PUFA levels alter the phospholipid membrane makeup of immune cells, which impacts pro-inflammatory signaling pathways [3], [6]. Shifting the balance from n−6 to n−3 PUFA exposure also decreases the production of pro-inflammatory eicosanoids from n−6 PUFA, and n−3 PUFA may directly interact with transcription factors such as NF-κB and PPAR-γ to downregulate the expression of inflammatory cytokines and other genes [3], [4]. Omega-3 PUFA may further regulate the immune response through the action of resolvins and protectins, anti-inflammatory lipid mediators that are biosynthesized from the n−3 PUFA eicosapentaenoic acid (EPA) and docosahexaenoic acid (DHA) [5], [9], [10]. Maternal dietary n−3 PUFA have been linked to increased resolvin and protectin levels in the placenta, suggesting a pathway by which n−3 PUFA improve pregnancy outcomes and inflammatory disease in children [8].

Beyond direct effects on immune cells and mediators, fatty acid intake can affect immunity through alterations in the gut microbiome. Current understanding on how dietary fats alter the microbiome include TLR4-dependent induction of local inflammation leading to altered host environment, shifts in immune cell membrane functions, and changes in nutrient availability favoring some organisms over others [11]–[13]. These alterations in the microbiota not only directly affect the host but can be passed onto the offspring.

We have previously shown that high saturated fat and n−6 intake by breeder mice resulted in altered microbiota in their offspring that heightened inflammatory responses and conferred increased susceptibility to models of autoimmune, allergic, and infectious diseases [13]. In this study, we sought to determine whether parental n−3 PUFA intake could also influence offspring microbiota and immune responses. Compared to offspring of mice fed a standard diet, we show that offspring of mice fed a high n−3 PUFA diet had an altered microbiome and decreased inflammatory responses in models of allergy and infection. These findings suggest that the reported anti-inflammatory effects of n−3 PUFA can persist in offspring of exposed mice, possibly through the inheritance of altered microbiota.

## Materials and Methods

All animal experiments were done in compliance with the guidelines of the National Institute of Allergy and Infectious Disease (NIAID) Institutional Animal Care and Use Committee. Our National Institutes of Health Institutional Animal Care and Use Committee (IACUC) approved this study and monitored the animals throughout.

### Dietary Exposure

**Figure 1:**
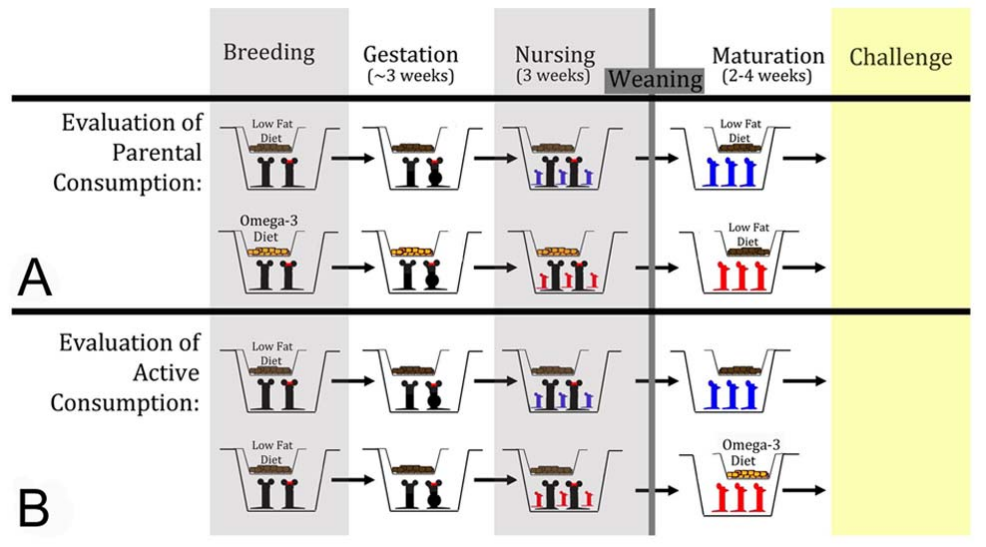
Summary of study design. (a) For experiments evaluating the effects of parental diet, littermate mice were placed on either Low Fat or high omega-3 formulations one day prior to being placed in breeding cages. Breeder mice were maintained on the different diets throughout gestation and nursing. When the pups were three weeks post-partum, they were weaned to new cages. All pups were weaned onto the Low Fat control diet. Two to four weeks after weaning, the mice were evaluated in the described models. (b) For evaluation of the effects of active diet consumption, the converse experiment was performed. Pups from breeders on the Low Fat control diet were weaned into new cages and placed on either the omega-3 or Low Fat control diet. Figure modified from [13].

We placed breeding mice on customized specialty diets with fatty acid content derived from natural oils (Figure 1, Table 1). The diets were derived from a master mix of proteins and micronutrients before the carbohydrates and dietary fats were added to ensure differences between diets were primarily in fatty acid content and the fat:carbohydrate ratio. Natural oils were added to our Low Fat control diet to achieve high n−3 content. Saturated fat content was also unavoidably increased by the use of natural oils, but, was far less than that used in our previous study investigating the effects of a high saturated fat Western diet [13]. All diet pellets were purchased from Research Diets Inc (New Brunswick, NJ) as previously described [13].

**Table 1:** Fatty acid content and source for the diets studied.

	Human RD	Omega-3	Low Fat
Protein (% kCal)	20	20	20
Carb (% kCal)	50	40	70
Fat (% kCal)	<30	40	10
% Fat Saturated	10	28	10
% Fat PUFA		46	72
% Fat MUFA		26	18
ω6 Source		Soy	Soy/Safflower
ω3 Source		Menhaden	Flaxseed
ω6: ω3 Ratio	2∶1	1∶2	2∶1

Breakdown of dietary components in the diets studied are shown, including protein, carbohydrates (carb), fat, and % of fat that was saturated, poly-unsaturated fatty acids (PUFA), or mono-unsaturated fatty acids (MUFA). The dietary source for each fatty acid is shown. Both diets were derived from natural oils. Human recommended diet (RD) reflects the guidelines of the United States Department of Agriculture. See Methods section for further details on diet formulation.

The breeders used in the study were all littermates and were placed on the high omega-3 (n−3) or control low fat (LF) diet one day before being placed in the breeding cages. Their pups were thus exposed to these diets *in utero* until birth, and for an additional 3 weeks via breast milk. At three weeks of age, the pups were weaned to new cages and all pups were placed on our LF control diet (pups from n−3 breeders were housed with littermates, but separately from the pups from LF breeders). After two to three weeks on the LF diet, the mice were placed into the challenge models described below and were maintained on the LF diet for the duration of each experiment. Therefore, at time of challenge, the only difference between the mice tested was the dietary fat and carbohydrate consumed by their parents during gestation and nursing. For investigation of the effects of actively being on a high n−3 diet, we placed mice on n−3 chow for two weeks after weaning from breeders on a standard diet. Of note, the high n−3 diet used in these experiments contains substantially higher amounts of n−3 than would be consumed in a human diet, and thus serves only to establish a proof-of-principle that high parental n−3 exposure can have effects in offspring.

### Mice

BALB/c mice were purchased from Jackson Labs, Bar Harbor, ME to set up breeders. Littermates were used as the breeders that were exposed to the experimental diets. Two to three breeder pairs per dietary group were maintained active at all times for approximately five months each. Mice were cared for as previously described [13].

### Escherichia Coli Sepsis

Mice were infected intraperitoneally with 10^4^ colony forming units of *E. coli* 01 K18 (gift from A. Cross, University of Maryland) and followed for two weeks for evidence of moribundity.

### Staphylococcus Aureus Infections

10^7^ CFU of USA300 LAC strain of MRSA (gift from F. DeLeo, Rocky Mountain Laboratories, NIAID, NIH) with Cytodex beads (Sigma, St. Louis, MO) were injected intradermally (100 µl) into the shaved back of each mouse. Resultant abscess size, bacterial burden, and skin cytokine analysis were done as previously described [13]. Taqman probes for IL-17A (Mm00439619_m1) and IL-1β (Mm01336189_m1) were purchased from Life Technologies. Comparison of signal was performed using the ΔΔCT (delta, delta Cycle threshold) method.

### Anaphylaxis and Antibody Measurements

Peanut oral sensitization and serologic evaluation of allergy was performed as previously described [13].

### Splenocyte and Colon Stimulation

Stimulation of spleen cells or colonic tissue with LPS was performed as previously described [13].

### Microbiome Analysis

DNA was extracted from sterilely excised cecal stool pellets from female BALB/c mice using QIAamp DNA stool mini kit (Qiagen). Quantitative analysis of 16S rDNA was performed as previously described on 1000–3000 sequences per sample using the established primer sequences [13]. Stool collection and sequencing were performed concurrently with those in the previously reported study [13] with overlapping low fat diet-exposed controls used in both studies. All microbiome sequence data were uploaded to the National Center for Biotechnology Information Sequence Read Archive database under accession number SRP026657; this can be accessed at http://www.ncbi.nlm.gov/Traces/sra/sra.cgi?study=SRP026657.

### Statistics

Means were compared using either two-tailed unpaired t test or ANOVA with Bonferroni’s post-test correction for multiple group comparison with Prism software (GraphPad, San Diego, CA). p values are designated as follows: NS or –, not significant; *, <0.05; **, <0.01; ***, <0.001; ****, <0.0001.

## Results

### High Omega-3 intake Altered Colonic Inflammation and Gut Microbiome

**Figure 2:**
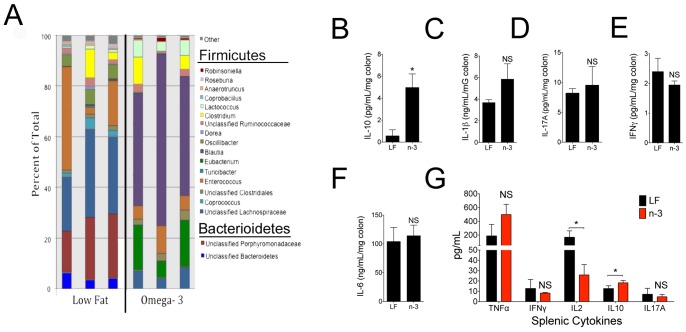
High omega-3 intake altered colonic inflammation and gut microbiome. (a) 16S ribosomal RNA genes in cecal stool samples of female mice. Each bar represents one mouse. Phyla (Bacterioidetes, Firmicutes, or other) are classified into genus or unclassified family members. Further breakdown of composition within each phylum can be found in Table 2. (b-f) Cytokine production from female excised colons stimulated with LPS for 24–72 hours (n = 4–6). (g) Cytokine production from male splenocytes stimulated with LPS for 24–72 hours (n = 4–6). Results are representative of 2–3 independent experiments in BALB/c mice and displayed as mean+s.e.m. Significance determined by t test. All experiments were repeated with similar results in both genders. Gender is indicated when representative experiments are shown; otherwise, data reflects both male and female mice with matched ratios within experiments. n designates number of mice per experiment. The results from the Low Fat group shown in (a) are a subset of what was previously reported in reference 13. The remaining data is novel.

In our previously published study, pups from breeders fed the Western diet (with high saturated and n−6 fat to carbohydrate ratio) had dramatic shifts in their gut microbiome compared to pups from mice fed a standard low fat diet [13]. Similar to those pups from breeders on a Western diet, pups in this study from our n−3 breeders had a loss of Bacteroidetes and an increased Firmicutes:Bacteroidetes ratio compared to pups from LF breeders (Fig. 2a and Table 1). This shift in composition has been correlated with deleterious inflammation [14], as was seen in our published studies on Western diet pups [13]. However, the pups from n−3 breeders did not show a heightened inflammatory response to LPS in the colon or spleen (Fig. 2b–g). Consistent with the reported anti-inflammatory effects of n−3 fatty acids, these pups had decreased IL-2 responses in the spleen and small but statistically significant increases in levels of the anti-inflammatory cytokine IL-10 in the colon and spleen (Fig. 2b and 2g). This discordant inflammatory effect despite similarly increased Firmicutes:Bacteroidetes ratio in pups from Western diet- and omega-3-fed breeders may reflect the differential presence of distinct members of these phyla. Omega-3 pups had significant increases in *Blautia, Oscillibacter, Clostridales, Robinsoniella, Lactococcus*, and *Eubacterium*, but a significant reduction in the frequency of *Lachnospiraceae, Anaerotruncus*, and *Roseburia* (Table 2). Therefore, similar to diets high in saturated and/or n−6 fat, high n−3 diets can also shift the gut microbiome, but each fatty acid composition promotes distinct microbiota and has unique effects on host inflammatory status.

**Table 2:** n−3 pups had altered microbiomes.

	LF1	LF2	LF3	n−3−1	n−3−2	n−3−3	Statistical comparison of groups
	(Percent of Total)						LF v Omega-3
**FIRMICUTES**	74.9	69.7	67.1	100	100	100	**
Lachnospiraceae	21.7	35.1	30.7	7.2	4.2	8.4	**
Eubacterium	0	0	0	17.7	6.6	18.5	*
Oscillibacter	4.5	6.1	5.8	0.5	0.5	0.3	***
Blautia	0.2	0.5	0.6	44.4	67.6	47.1	**
Turicibacter	0	0.5	0.2	0	0	0	–
Enterococcus	40.7	2.7	17.6	5.4	10.8	5.6	–
Unclassified Clostridiales	1.3	1.6	1.9	2.3	3.1	4	*
Coprococcus	1.2	4.2	2.2	0.1	0	0	*
Dorea	0.6	1.2	0.4	0	0	0	–
Ruminococcaceae	2.2	3.3	1.5	3	0.8	2.6	–
Clostridium	0	11.5	3	10.7	0	5.5	–
Lactococcus	0.6	1.3	1	6.8	3.9	6.1	**
Coprobacillus	0.8	0	0.4	0	0	0	–
Anaerotruncus	0.9	0.9	0.9	0.1	0	0	***
Roseburia	0.2	0.7	0.8	0	0	0	*
Robinsoniella	0	0.1	0.1	0.7	1.7	0.8	*
**BACTERIODETES**	22.6	27.9	29.4	0	0	0	**
Porphyromonadaceae	16.3	24.4	25.2	0	0	0	**
Unclassified Bacteroidetes	6.3	3.5	4.2	0	0	0	**
**OTHER**	2.5	2.4	3.5	1.1	0.8	1.1	**
Firmicute:Bacteroidetes	3.3∶1	2.5∶1	2.3∶1	>1e9∶1	>1e9∶1	>1e9∶1	***
Shannon Diversity Index	1.760	1.993	1.958	1.753	1.225	1.696	–

16S ribosomal RNA genes in cecal stool samples as percent of total yield. Pups from indicated breeder diets were weaned to LF diet either in cages with their littermates. Each column represents one mouse. The column order of mice mirrors the presentation in Figure 2a. LF, Low Fat; n−3, omega-3. Results are representative of 2 independent experiments. Significance determined by t test (phyla) or ANOVA with Bonferroni’s correction (genera): * = p value <0.05, ** = <0.01, *** = <0.001, – = not significant. The results from the Low Fat group shown in this table are a subset of what was previously reported in reference 13. The remaining data is novel.

### High Omega-3 intake Altered Allergic Responses

**Figure 3:**
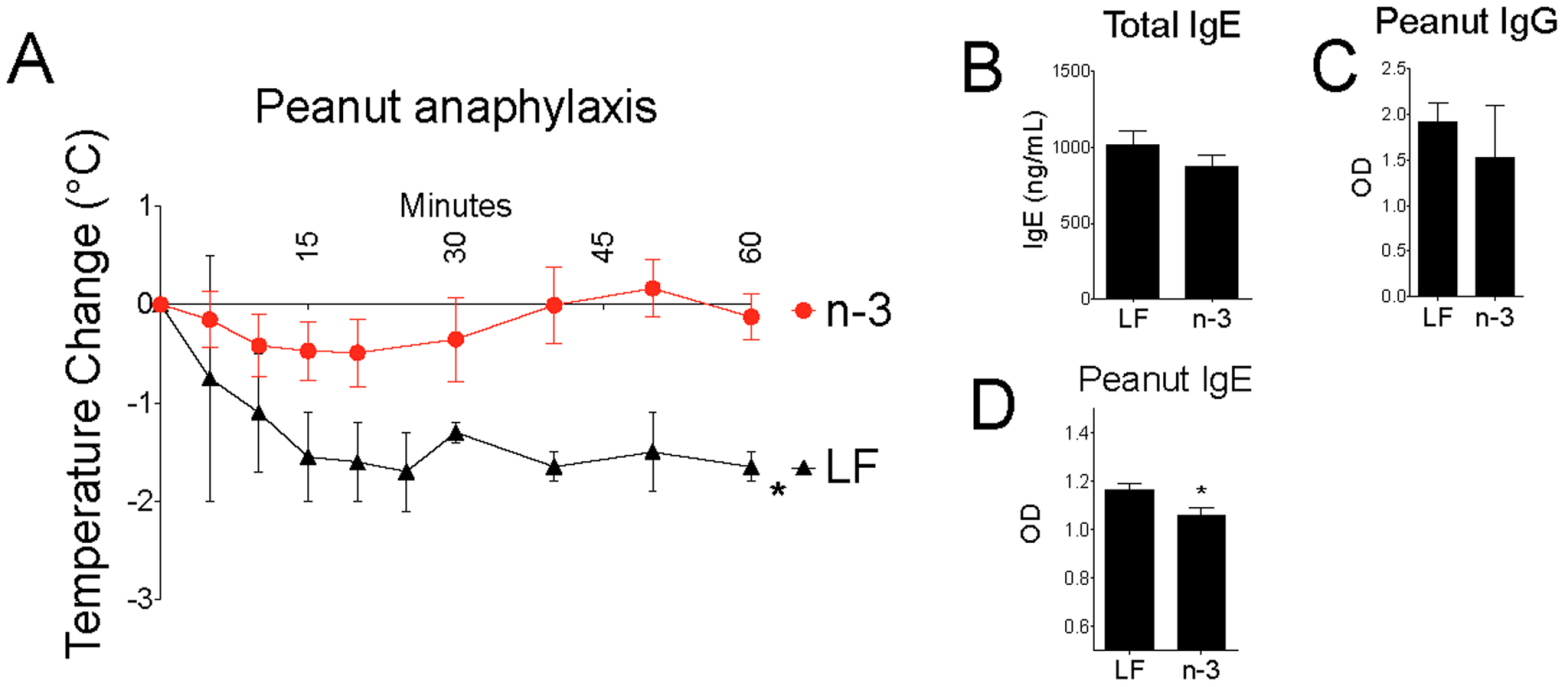
High omega-3 intake altered allergic responses. (a–d) Weaned male BALB/c pups were gavaged with peanut protein and cholera toxin weekly for 4–8 weeks before challenge. Temperature drop after intraperitoneal challenge (a), total IgE (b), and peanut-specific antibodies (c and d) (n = 5). LF, Low Fat; n−3, Omega-3 diet. Results are representative of 2 or more (b-d) or combined from two (a) independent experiments and displayed as mean+s.e.m. Significance determined by t test. n designates mouse number per group per experiment.

Pups from n−3 breeders were protected from the temperature drop seen in LF pups after challenge in a model of peanut anaphylaxis (Fig. 3a). This phenomenon was seen despite no significant alterations in total IgE or peanut-specific IgG and a small but significant difference in peanut-specific IgE (Fig. 3b–d). These results are consistent with prior research showing n−3 fatty acids protecting against sensitization to milk in a mouse model [15], inhibiting the release of the pro-allergic interleukins IL-4 and IL-13 from human mast cells [16], and being associated with reduced incidence of asthma in children [17].

### High Omega-3 intake Inhibited Control of Methicillin-resistant S. aureus (MRSA) Skin Infection

The inhibition of inflammatory responses by n−3 PUFA intake may hinder the immune responses required to control infectious agents. Indeed, prior research has suggested that n−3 fatty acids were detrimental in Gram-negative sepsis [18].

**Figure 4:**
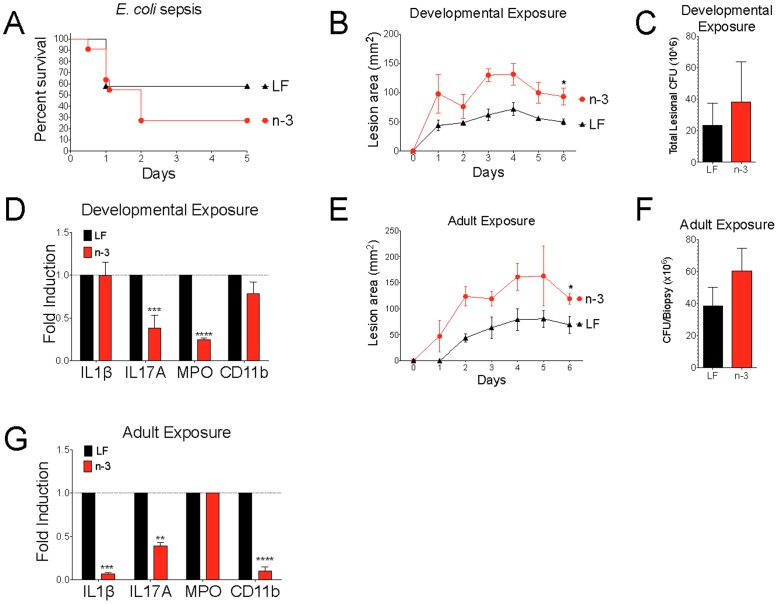
Omega-3 consumption altered MRSA susceptibility. (a) Survival after infection with *E. coli* 01 K18 in pups of breeders exposed to LF or n−3 diet (n = 10–12). (b–g) *Staphylococcus aureus* (MRSA USA300) skin infection in male BALB/c mice. Lesion sizes (b), CFU (c), and mRNA expression in skin abscess tissue normalized against LF controls (dotted line) (d) (n = 5–6) in offspring mice. Lesion sizes (e), CFU (f), and mRNA expression in skin abscess tissue normalized against LF controls (dotted line) (g) (n = 10) in adult male mice. Results are representative of two (d and g) or combined from 2–3 (a–c and e–f) independent experiments and displayed as mean+s.e.m. Significance determined by t test (b–c, e–f), ANOVA with Bonferroni’s correction (d and g), or Kaplan-Meier (a). n designates mouse number per group.

Compared to pups from LF breeders, pups from n−3 breeders in our study had a trend toward increased fatality after *E. coli* challenge that did not reach statistical significance (Fig. 4a). Interestingly, in a model of MRSA skin infection, pups from n−3 breeders had larger lesion sizes during skin infection with MRSA (Fig. 4b). Although bacterial load in the skin was not significantly altered (Fig. 4c), the n−3 pups did have evidence of an impaired inflammatory response with reduced skin IL-17A mRNA induction (Fig. 4d) and reduced markers of neutrophil infiltration as measured by myeloperoxidase and CD11b mRNA levels in the skin (Fig. 4d). Active exposure to the high n−3 diet in adult mice also led to similarly larger lesion sizes (Fig. 4e), non-significant effects on CFU (Fig. 4f), and reduced IL-1β and IL-17A cytokine responses (Fig. 4g). Taken together, these results are consistent with direct anti-inflammatory effects of n−3 exposure during active consumption but further highlight the potential of these effects to be transmitted to offspring.

## Discussion

Our previous work illustrated the impact of a diet high in both saturated and omega-6 fatty acids on the immune development of offspring after exposure *in utero* and during nursing, identifying microbiota alterations that heightened deleterious inflammatory responses in models of autoimmunity, allergy, and infection [13]. Here we report the effects of parental exposure to a high n−3 diet and found that this resulted in a distinctly altered microbiome in the offspring. The effects on offspring susceptibility to allergic disease and infection were modest, but suggested promotion of anti-inflammatory responses that would be consistent with speculations that low n−3 exposure in modern diets may contribute to increasing prevalence of inflammatory disorders.

The mechanisms by which offspring immunity is altered by parental n−3 exposure remain incompletely elucidated. The reported ability of n−3 intake to induce resolvins and protectins [7], [9], [10], [19] would fit with our observed decreases in inflammatory cytokine responses and neutrophil recruitment, but would most logically be occurring in directly exposed mice. However, developmental exposures regulating production of resolvins and protectins are also incompletely understood and may contribute to the observed effects. Detailed analyses of tissue levels of these compounds and/or resolvin and protectin knockout mice would be needed to fully evaluate the role of these mediators in both pups and directly exposed mice. Additionally, omega-3 intake alters dendritic cell activity through reductions in phagocytosis, enhancement of anti-inflammatory DC subtypes, reduced T cell activation, and inhibited TNFα production [20], [21] and TLR4 responsiveness [12]; evaluation into these effects on both the pups and parents will be an interesting area for future study.

Alterations in regulatory T (Treg) function and IL-10 production may be another potential explanation for our observed phenotype, especially given results in sites distant from the colonic biome such as the skin and intraperitoneal cavity. Alterations in Treg cells and/or reduction in IL-10 levels can profoundly affect inflammation [22], susceptibility to skin infections with *Staphylococcus* [23], development of allergy [24], and control of sepsis [25]. Furthermore, given alterations in the gut microbiome can directly alter Treg cells development [26], the systemic effects of colonic biome changes may conceivably be mediated through changes in Treg development. While IL-10 is only a surrogate for Treg frequency [27], it seems plausible that our observed changes in IL-10 production in the colon (Fig. 2B) and spleen (Fig. 2G) may represent similar systemic alterations in Treg function in our mice. Omega-3 enhancement of Treg cells may explain the protection from allergic disease and reduced inflammatory response. However, our results with MRSA skin infection are inconsistent with human findings indicating that increased Treg function is protective against *Staphylococcus* [28]. This may be related to n−3 induced alterations in resolvins, protectins, or other competing pathways. Further detailed evaluation of Treg number and function in the colon, skin, and spleen of both our n−3 pups and parents will therefore be an important area of future evaluation.

In our previous studies on the effect of high saturated fat diet exposure [13], co-housing studies suggested that reversible alterations in the pup microbiome drove observed immunologic and disease susceptibility phenotypes. In light of the known transmissibility of maternal microbiota to offspring during parturition and possibly even *in utero* [11], [13], [29]–[31], it seems likely that this mechanism may also account for the observed shifts in n−3 pup microbiota and contribute to the observed immunological phenotypes. Since we did not perform co-housing experiments in this work, the prediction that our findings are secondary to the gut microbiome must only be considered an extrapolation from prior studies and established literature. Furthermore, comparison of the microbiomes of pups from n−3 breeders to mice directly fed after weaning will help elucidate the stability of the biome after birth. Interestingly however, our microbiome data suggested a predominant increase in *Blautia*, a genus whose absence has been associated with various inflammatory disorders [32]–[38]; the overlap of *Blautia*, inflammation, and our findings supports a plausible connection of our results to the gut biome. However, identification of specific bacteria that promote or inhibit specific immunological responses will require substantial further investigation and further studies employing fecal transplant of isolated taxa will be needed to elucidate mechanisms to alter the gut microbiome for therapeutic effects.
